# Forward and Reverse Genetics of B Cell Malignancies: From Insertional Mutagenesis to CRISPR-Cas

**DOI:** 10.3389/fimmu.2021.670280

**Published:** 2021-08-13

**Authors:** Joanna C. Dawes, Anthony G. Uren

**Affiliations:** ^1^Medical Research Council, London Institute of Medical Sciences, London, United Kingdom; ^2^Institute of Clinical Sciences (ICS), Faculty of Medicine, Imperial College London, London, United Kingdom

**Keywords:** B cell leukemia, B cell lymphoma, mouse models, insertional mutagenesis, exome sequencing, reverse genetics, CRISPR-Cas, shRNA****

## Abstract

Cancer genome sequencing has identified dozens of mutations with a putative role in lymphomagenesis and leukemogenesis. Validation of driver mutations responsible for B cell neoplasms is complicated by the volume of mutations worthy of investigation and by the complex ways that multiple mutations arising from different stages of B cell development can cooperate. Forward and reverse genetic strategies in mice can provide complementary validation of human driver genes and in some cases comparative genomics of these models with human tumors has directed the identification of new drivers in human malignancies. We review a collection of forward genetic screens performed using insertional mutagenesis, chemical mutagenesis and exome sequencing and discuss how the high coverage of subclonal mutations in insertional mutagenesis screens can identify cooperating mutations at rates not possible using human tumor genomes. We also compare a set of independently conducted screens from *Pax5* mutant mice that converge upon a common set of mutations observed in human acute lymphoblastic leukemia (ALL). We also discuss reverse genetic models and screens that use CRISPR-Cas, ORFs and shRNAs to provide high throughput *in vivo* proof of oncogenic function, with an emphasis on models using adoptive transfer of *ex vivo* cultured cells. Finally, we summarize mouse models that offer temporal regulation of candidate genes in an *in vivo* setting to demonstrate the potential of their encoded proteins as therapeutic targets.

## Introduction

**Figure 1:**
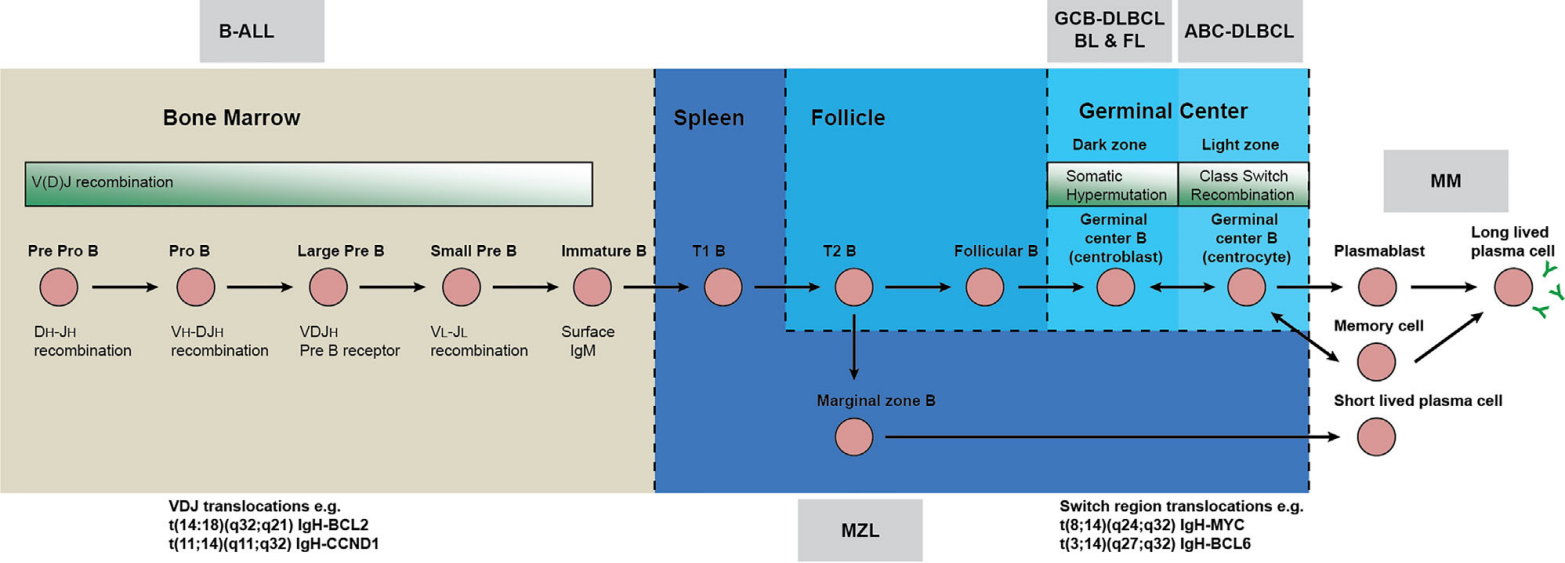
Neoplastic mechanisms throughout B cell development. Early B-cell development in adult bone marrow is characterized by generation of the B-cell receptor (BCR). Immunoglobulin heavy chain (IgH) gene rearrangements (V_H_, D_H_ and J_H_), together with the V_L_-J_L_ rearrangements of light chain (IgL) gene segments generate a B-cell repertoire of antibodies that recognize a variety of antigens. If the BCR does not form correctly, or is autoreactive, the cell undergoes apoptosis or receptor editing. Immature B-cells with functional BCRs, migrate to secondary lymphoid organs like the spleen and lymph nodes where they form follicles during the T-cell dependent immune response. Within the follicle, B-cells undergo two further remodeling steps in the germinal centers (GC): somatic hypermutation (SHM) and class switch recombination (CSR). SHM introduces point mutations, deletions or duplications into the variable region of the immunoglobulin genes. CSR replaces the IgH chain constant region, altering the effector response but not the antigen binding domain. GC B-cells that have undergone CSR and have high affinity IgG BCRs are selected for and differentiate into plasma cells and long-lived plasma cells, the effectors of adaptive immunity. The assignment of translocations to either V(D)J recombination in pro/pre B cells in the marrow or class switch recombination in the germinal center is based on the location and sequences of junctions present in each translocation. For instance, V(D)J derived translocations typically give rise to *BCL2* or *CCND1* translocations to the Ig variable regions whereas class switching frequently results in translocations between *BCL6* or *MYC* and Ig constant regions.

**Figure 2:**
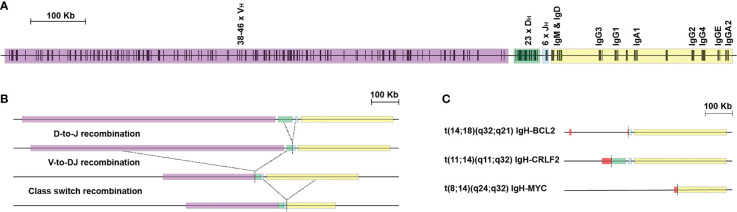
Aberrent V(D)J & class switch recombination creates oncogenic translocations. Immunoglobulin variable region rearrangement and class switch recombination are error prone processes. **(A)** The immunoglobulin heavy chain locus is divided into different repetitive elements including variable segments (VH purple), diversity segments (DH green), joining segments (JH blue) and constant regions (yellow). Vertical bars depict both functional gene segments (typical numbers are indicated), non-functional pseudogenes (numbers not indicated) and repetitive elements (adapted from imgt.org). **(B)** Expression of recombinase activating genes (RAG) during pre pro, pro and pre B stages creates breaks between V(D)J segments, which are resolved by excision of intervening DNA. Activation induced cytidine deaminase (AID) deaminates cytosine residues of single stranded DNA which are exposed during transcription. The resulting mismatch is processed by error prone DNA repair mechanisms (including endonuclease G) resulting in excision of the default IgM/IgD constant region and fusion of the joining segments with constant regions of other isotypes. **(C)** Errors in resolving breaks initiated by RAG and AID can resolve as translocations with non-immunoglobulin loci. Break points adjacent to the heavy chain J_H_ or D_H_ segments indicate that translocations occur at the pre pro B cell stage in the bone marrow (*IgH/BCL2* or *IgH/CRLF2*). Similar translocations also originate from immunoglobulin light chain loci. Translocations may also result from class-switch recombination in the light zone of the germinal center, evidenced by breakpoints in the immunoglobulin constant region (*IgH/Myc*). Breakpoints adjacent to successfully rearranged V(D)J segments with somatically mutated variable portions indicate that an AID mediated translocation has occurred later in development in the germinal center dark zone.

B cell neoplasms can be categorized by their cell of origin, each subtype being representative of a discrete stage in differentiation with characteristic phenotypes and genetic lesions (**Table 1**, **Figure 1**) [reviewed in (1, 2)]. Accurate modelling of these diseases in mice requires alteration of various biological processes at different stages of B cell development including differentiation, migration, rearrangement of immunoglobulin genes, T helper interactions, positive selection for antigen and negative selection against autoreactivity. Like most tumors, B cell malignancies accumulate amplifications, deletions, rearrangements, deregulation of methylation and non-synonymous point mutations. Additionally, B cells are characterized by remodeling of the immunoglobulin loci by recombinase activating gene (RAG) mediated V(D)J recombination of immunoglobulin variable regions and by activation induced cytidine deaminase (AID) mediated class switch recombination. Errors of these processes mean immunoglobulin loci are the most frequent translocation partners of B cell malignancies, often placing oncogenes under the control of the highly expressed immunoglobulin promoters and enhancers and causing deregulated and constitutive expression (3–6) (**Figure 2**). Somatic hypermutation by AID is also a known source of oncogenic mutations in B neoplasms as evidenced by the mutation fingerprint of commonly mutated genes, and aberrant somatic hypermutation of non-immunoglobulin genes at hotspots located throughout the genome also contributes to lymphomagenesis (4, 7–9).

**Table 1:** Major subtypes of B cell malignancies.

Subtype	Nearest normal B cell phenotype
Burkitt lymphoma (BL)	Germinal Center B cell
B-cell acute lymphoblastic leukaemia (B ALL)	Pre B cell, Pro B cell, Mature B cell
Chronic Lymphocytic Leukemia (CLL), Small Lymphocyte Lymphoma (SLL)	Mature B cell or Post–germinal center B cells
Diffuse large B-cell lymphoma (DLBCL)	Activated B cell or Germinal Center B cell
Follicular Lymphoma (FL)	Germinal center B cell
Hairy cell leukemia (HZL), variant hairy cell leukemia (HZL-v)	Marginal zone/memory B cells
Hodgkin lymphoma (HL)	Germinal Center B cell
Lymphoplasmacytic Lymphoma (LPL), Morbus Waldenström	Plasma cells
Mantle cell lymphoma (MCL)	Mantle B cell
MALT lymphoma (MALT)	Post Germinal Center B cells
Marginal zone lymphoma (MZL)	Marginal zone B cells
Monoclonal B-cell lymphocytosis (MBL)	Germinal Center B cell
Plasma cell myeloma (PCM), Multiple Myeloma (MM), Monoclonal Gammopathy of undetermined significance (MGUS)	Plasma cells
Prolymphocytic leukemia (B-PLL)	Pro B cells (aggressive CLL variant)

Speculation on the developmental stage that each malignancy is derived from is based on a combination of genetic lesions and phenotypic characteristics [reviewed in (1, 2)]. The cell of origin suggested by translocation breakpoints does not always match the cell of origin suggested by somatic hypermutations and/or markers expressed on the cell surface. Follicular lymphoma (FL) and diffuse large B cell lymphoma (DLBCL) appear to have a mature B cell phenotype. Nonetheless, they share a common translocation *IgH/BCL2* t(14;18)(q32;q21) which occurs early in B cell development during V(D)J recombination mediated by RAG in the primary lymphoid organs (10). This translocation places the anti-apoptotic *BCL2* gene under control of the immunoglobulin heavy chain (*IgH*) enhancer and plays a causal role in germinal center derived malignancies. Though this translocation is commonly an initiating event in development of FL, it alone is not sufficient to initiate lymphomagenesis and similar translocations can be detected in the peripheral blood and lymph nodes of healthy individuals (11). Similarly, whilst chronic lymphocytic leukemia (CLL) is also considered a mature B cell malignancy, the propensity for cells to become malignant appears to arise as early as the hematopoietic stem cell (HSC) stage and driver lesions are detectable in the hematopoietic progenitors of some patients (12–14). CLL is also characterized by distinct subtypes that either have unmutated variable regions or hypermutated post germinal center variable regions. Mantle cell lymphoma (MCL) has a similar bifurcation between mutated/unmutated variable regions.

Further complicating matters, some B cell neoplasms result from the transformation of non-malignant monoclonal outgrowths or from other neoplasm subtypes. DLBCL can arise from the transformation of CLL, small lymphocytic leukemia (SLL) or FL (15) or less commonly from mucosa associated lymphoid tissue (MALT) lymphoma (16, 17) or Hodgkin lymphoma (HL) (18). CLL can itself develop from the precursor condition monoclonal B cell lymphocytosis (19) and a high proportion of multiple myeloma (MM) arise from monoclonal gammopathy of undetermined significance (20, 21).

Recent genomic studies of B cell malignancies implicate a handful of well-characterized, commonly mutated or translocated genes as well as a long tail of genes that have limited or no experimental evidence to support a role in disease. Since it is unlikely that all these mutations are driving oncogenesis there is need for validation methods such as functional genomic screens, forward genetic screens and comparative genomics to scrutinize driver mutations and better prioritize candidates for further study. The diverse mechanisms by which mutations contribute to disease means that many can only be tested within the context of specific developmental stages, predisposing mutations and optimal antigen receptor stimulation. For secondary/later stage mutations, oncogenic function and selection may only occur within a specific context of germline variants and/or early stage initiating mutations. This exponentially expanding set of parameters for validation experiments therefore requires prioritization by analysis of mutation profiles and higher throughput testing methods.

In some respects, sequencing human tumors can be viewed as a special case of forward genetics, i.e. surveying genetic diversity under selection in order to identify which genetic changes drive a biological process. Forward genetic screens can also be targeted to the somatic cells of mice using either chemical or insertional mutagens. In the context of mouse cancer models, reverse genetics is the introduction of variation known to be associated with cancer in order to study the function of that variation. This variation can be introduced to the germline or targeted to specific populations of somatic cells.

The benefit of using mouse models to study hematologic malignancies is that the major genes and biological processes driving hematopoiesis and immunity are sufficiently conserved such that human malignancies can be recapitulated by the introduction of equivalent mutations. Mice also allow the complexity of immune system biology to be recapitulated *in vivo* in ways not yet possible with cultured cells or organoids.

This review covers mouse models used to rapidly screen for and validate large numbers of candidate genes driving B cell malignancies and is aimed at readers seeking to understand various techniques for targeting different developmental stages and disease subtypes. We provide a history of forward and reverse genetic approaches in mice that balance the trade-offs between fidelity, precision, and throughput. In the first half we discuss how forward genetics in mice can independently validate the oncogenic function of rarely mutated genes from human studies, and in some cases is a tool leading to discovery of human cancer genes. In the second half we cover reverse genetic screens and validation experiments where throughput is increased through the use of transplantation of primary cells transduced with ORF, shRNA and CRISPR-Cas viral constructs. Throughout we also emphasize strategies to direct models toward specific malignancy subtypes by a combination of germline lesions, targeted cell populations and temporal control of gene expression.

## Forward Genetic Models

Many driver genes are difficult to identify because they are subject to deregulation by non-coding mutations, copy number changes or epigenetic modification. Comparative genomic analyses of human tumors with the findings from forward genetic screens in mouse models can narrow down the identity of these driver genes. Mice with germline mutations that sensitize them to a specific disease subtypes are subjected to mutagenesis of their somatic cells and the resultant tumors are sequenced. We discuss here the combined use of genetically modified mice, insertional mutagens and exome sequencing as tools to perform forward genetic screens in B cell neoplasms (summarized in **Table 2**). Observing which mutations are present in both mouse and human cohorts, or which mutation combinations are selected to co-occur more or less frequently than expected by chance can prioritize validation experiments.

**Table 2:** Forward genetic screens in mouse models of B cell malignancies.

Publication	Study type	Mutagen	Mouse strains	B cell malignancies
van Lohuizen et al. (22)	MuLV mutagenesis	Mo-MuLV	Eμ-Myc	pre B cell lymphoma
Haupt et al. (23)	MuLV mutagenesis	Mo-MuLV	Eμ-Myc	pre B & mature B cell lymphoma
Shinto et al. (24)	MuLV mutagenesis	Mo-MuLV	Eμ-BCL2	pre B & B cell lymphoma
van der Lugt et al. (25)	MuLV mutagenesis	Mo-MuLV	Eμ-Myc Pim1-/-	pre B cell lymphoma
Sheppard et al. (26)	MuLV mutagenesis	Mo-MuLV	Eμ-Mycn	pre B cell lymphoma
Mikkers et al. (27)	MuLV mutagenesis	Mo-MuLV	Eμ-Myc; Pim1-/-; Pim2-/-	pre B cell lymphoma
Dang et al. (28)	MuLV/ENU mutagenesis/exome sequencing	Mo-MuLV or ENU	thymectomized Pax5+/- & Pax5-/-	pre B leukemia / B cell leukemia
Webster et al. (29)	MuLV mutagenesis	Mo-MuLV	Eμ-BCL2, Vav-BCL2	mature B cell lymphoma
Martín-Hernández et al. (30)	MuLV mutagenesis	Akv1-99 MuLV	NMRI mice	B cell lymphoma
Ma et al. (31)	MuLV mutagenesis	Akv MuLV & derivatives	NMRI mice	DLBCL and plasmacytoma
Sorensen et al. (32)	MuLV mutagenesis	Akv MuLV & derivatives	NMRI mice	B cell lymphoma, plasmacytoma
Liu et al. (33)	MuLV mutagenesis	Akv MuLV & derivatives	NMRI mice	B cell lymphoma, plasmacytoma
Pyrz et al. (34)	MuLV mutagenesis	Akv MuLV & derivatives	NMRI mice	B cell lymphoma
Hartley et al. (35)	MuLV mutagenesis	endogenous ecotropic MuLV	NFS.V+ mice	DLBCL, BL, MZL, FL, SLL & Immunoblastic lymphoma
Suzuki et al. (36)	MuLV mutagenesis	endogenous ecotropic MuLV	AKXD mice	DLBCL, BL, MZL, FL, Pre B & Immunoblastic lymphoma
Tsuruyama et al. (37)	MuLV mutagenesis	endogenous ecotropic MuLV	SL/Kh mice	pre B cell lymphoma / B cell lymphoblastic lymphoma
Jin et al. (38)	MuLV mutagenesis	endogenous ecotropic MuLV	SL/Kh mice	pre B cell lymphoma / B cell lymphoblastic lymphoma
Shin et al. (39)	MuLV mutagenesis	endogenous ecotropic MuLV	NFS.V+ mice	Splenic marginal zone lymphoma
Suzuki et al. (40)	MuLV mutagenesis	endogenous ecotropic MuLV	AKXD-Blm m3 mice	B cell lymphoma
Weiser et al. (41)	MuLV mutagenesis	endogenous ecotropic MuLV	AKXD mice	pre B cell & B cell leukemia/lymphoma
Tsuruyama et al. (42)	MuLV mutagenesis	endogenous ecotropic MuLV	SL/Kh mice	pre B cell lymphoma / B cell lymphoblastic lymphoma
Van Der Weyden et al. (43)	SB mutagenesis	ETV6 knock in SB	ETV6-RUNX1-SB knock in	B cell precursor ALL
Zanesi et al. (44)	SB mutagenesis	SB	Emu-Tcl1	CLL
Van Der Weyden et al. (45)	SB mutagenesis/exome	ETV6 knock in SB	ETV6-RUNX1-SB knock in; Pax5+/-	B cell precursor ALL
Heltemes-Harris et al. (46)	SB mutagenesis	SB	Stat5b-CA	B ALL
Rahrmann et al. (47)	SB mutagenesis	SB	Trp53^R270H^ or Pten +/-	FL and DLBCL
Weber et al. (48)	PB mutagenesis	Rosa26 knock in PB	*Blm*m3 mice	DLBCL
Sander et al. (49)	exome	none	Myc and PI3K conditional	BL
Sungalee et al. (50)	exome of premalignant cells	none	Eμ-hBCL2 transduced mice	FL & FL in situ
Gough et al. (51)	exome	none	Vav-NUP98-PHF23	progenitor B-1 ALL
Martin-Lorenzo et al. (52)	exome	none	Pax5 +/-	precursor B ALL
Dang et al. (28)	MuLV/ENU mutagenesis/exome sequencing	Mo-MuLV or ENU	Pax5 +/-	pre B leukemia / B cell leukemia
Van Der Weyden et al. (45)	SB mutagenesis/exome	ETV6 knock in SB	ETV6-RUNX1-SB knock in; Pax5+/-	B cell precursor ALL
Duque-Afonso et al. (53)	exome	none	E2A-PBX1 conditional	B ALL
Lefebure et al. (54)	exome	none	Eμ-Myc	BL
Rodríguez-Hernández et al. (55)	exome	none	Sca1-*ETV6-RUNX1*	precursor B ALL
Gough et al. (56)	exome	none	Vav-NUP98-PHF23	progenitor B-1 ALL
Mouly et al. (57)	exome	none	Tet2 +/- and Tet2 -/-	B cell lymphoma
Jamrog et al. (58)	exome	none	PAX5-ENL knockin	B ALL
Zaborsky et al. (59)	exome	none	Emu-TCL1	CLL
Flümann et al. (60)	exome	none	Myd88 & Bcl2 conditional	DLBCL
Vicente-Duenãs et al. (61)	exome	none	Pax5+/-	B ALL

### Insertional Mutagenesis Screens in Somatic Cells

Somatic insertional mutagenesis screens are one of the most efficient tools for performing forward genetic screens in mouse models of cancer. When insertional mutagens are integrated randomly throughout the genome of somatic cells, they can deregulate and disrupt genes in a manner analogous to chromosomal rearrangements, deletions, non-coding mutations and truncating coding mutations. Mouse tissues are typically mutagenized by replicative retroviruses or germline copies of transposons mobilized in different tissues. Under optimal conditions a subset of these mutagenized cells eventually give rise to malignancies.

The primary benefit of analyzing integration mutations is the ease with which they can be mapped to the genome by amplifying sequences that flank the integration using various ligation mediated PCR methods. In analyzing cohorts of tumors in these screens, loci found to have insertion sites in independent tumors, more frequently than expected by chance, are defined as common insertion sites or common integration sites (CISs). Selection of mutations at these CIS loci indicates that they cause changes in expression levels or cause disruption/truncation of cancer drivers. There can be substantial phenotypic and genetic variability between malignancies of a single cohort and driving specificity toward B cells rather than T or myeloid lineages has been achieved through a combination of limiting mutagenesis to B lineages, screening mice with a predisposition toward B malignancy subtypes and curating uniform subsets of B lineage tumors from mixed cohorts.

### Retroviral Mutagenesis in the Hematopoietic Compartment

Slow transforming retroviruses have an extensive history of use as insertional mutagens in the hematopoietic compartment [reviewed in (62)]. When newborn mice are infected with slow transforming retroviruses, they fail to mount an immune response and consequently develop a viremia that lasts the lifetime of the animal. Successive rounds of reinfection lead to an accumulation of insertion mutations in cells of the hematopoietic compartment, where the high rate of proliferation during early postnatal development makes them the preferred host cells for virus propagation. In some strains the spontaneous activation of endogenous ecotropic retroviruses gives rise to disease by a similar process. Over time, mutations providing a selective advantage will lead to the clonal expansion of cells with multiple oncogenic insertion mutations.

These viruses induce a range of hematologic malignancies. B cell tumor cohorts have been generated by choosing specific combinations of virus strain and host mouse strain. Sequences within the virus LTRs are responsible for cell type-specific expression and alterations can skew tumors toward B cell subtypes (63, 64). Akv is an endogenous, ecotropic murine leukemia virus isolated from the AKR strain which causes mature B cell lymphomas with an FL and DLBCL phenotype (65). Mutation of the Akv LTR enhancer sequences causes plasmacytoma like disease (66) and mutation of splice sites broadens the diversity of B malignancy subtypes (32). Somatic reactivation of endogenous ecotropic proviruses can also give rise to B lineage malignancies in inbred mouse strains (SJL/J mice, CWD/LeAgl, SEA/GnJ, SL/Hk) (67, 68), recombinant inbred strains (AKXD) (69) and the NFS.V+ congenic mice (bearing ecotropic MuLV loci from AKXD or C58/Lw) (70, 71). When integration sites from B malignancies of these models were cloned, many were found recurrently at sites that were known or have subsequently been found to be drivers of B cell malignancies (27, 30–39, 41, 42) (**Table 2**).

Retroviral integrations tend to increase the expression of oncogenes although occasionally intragenic integrations cause loss of function of tumor suppressors. One of the more innovative strategies to increase the proportion of tumor suppressors identified is the screening of mice on a *Blm* hypomorphic background (40). *Blm* encodes a recQ DNA helicase the loss of which creates genomic instability. This encourages DNA repair by homologous recombination, which in turn leads to increased sister chromatid exchange and large stretches of loss of heterozygosity. When integration of a virus disrupts a tumor suppressor gene, any subsequent deletions or loss of heterozygosity that removes the wild type copy will increase selection of tumor suppressor mutations.

Enforced expression of B cell lymphoma oncogenes such as *MYC*, *NMYC* or *BCL2*, in the B cell compartment or loss of tumor suppressors can also skew MuLV driven malignancies toward B cell lymphoma and leukemias (22–26). Recent large scale analyses of the common integration sites of two *BCL2* transgenic strains infected with Moloney MuLV (MoMuLV) shows a statistically significant bias toward verified drivers of human B lymphoma and leukemia (*Pou2f2*, *Pax5*, *Ikzf3*, *Ebf1*) in addition to dozens of other candidate loci (29).

### Transposon Mutagenesis in the Hematopoietic Compartment

The use of DNA transposons as somatic insertional mutagens in mice has led to dozens of tissue and cell type specific screens [reviewed in (72–74)]. The Sleeping Beauty (SB) transposon isolated from salmonid fish and the piggyBac (PB) transposon isolated from the cabbage looper moth have both been adapted for use in mammalian cells. When mice bearing a concatemer of a transposon as a germline transgene are crossed to mice that express the cognate transposase, this causes mobilization of the transposon in somatic cells. By controlling expression of the transposase and the cargo of the transposon (typically a promoter trap and/or gene trap) this technique generates a wider spectrum of tumor types than slow transforming retroviruses, although this versatility is tempered by several caveats. Transposons have a tendency to hop in *cis* which can complicate analysis of integrations near the initial concatemer. Furthermore, remobilization of the transposon during tumor development can cause local hopping around the region of tumor initiating mutations and there is typically a higher level of background non-CIS mutations that provide no selective advantage to tumor cells. The screens discussed in detail below have successfully targeted transposon mutagenesis to the B cell compartment using a combination of predisposing mutations combined with tissue specific transposase expression.

Weakly oncogenic mutations observed in human tumors do not always lead to tumor development in mouse models due to a lack of cooperating mutations. One of the first B cell malignancy SB screens used a mouse model of the t(12;21)(p13;q22) *ETV6/RUNX1* (*TEL/AML1*) fusion transcript, seen frequently in B cell acute lymphoblastic leukemia (ALL). Mice expressing the *ETV6/RUNX1* fusion and the SB transposase variant HSB5 from the endogenous *Etv6* locus had a background of long latency hematologic malignancies similar to wild type controls i.e. the fusion itself caused only slightly higher rates of lymphomagenesis (43). When this allele was combined with a gene trap/promoter trap transposon the mice developed a spectrum of acute myeloid leukemia (AML), T cell ALL and B cell precursor ALL. By sequencing the integrations of B ALL samples, recurrent truncating mutations were observed in *Ebf1*, a tumor suppressor of B ALL (75, 76) which encodes a transcriptional activator of another B cell ALL tumor suppressor *Pax5*.

*STAT5* activating mutations are observed in a small proportion of B ALL, however mice expressing a constitutively active form of *STAT5B* (*STAT5*-CA N642H) do not develop ALL (46). Combining this allele with a transposon gene/promoter trap concatemer, a Cre-inducible SB and *Cd79a*-Cre (switching in developing B cells) induced progenitor B cell leukemias thereby verifying *STAT5B* N642H as an oncogene in B ALL. Integrations of 65 mice identified 12 CIS, the most commonly mutated genes suggesting three major mechanisms for *STAT5* mediated transformation: disruption of B-cell development (*Sos1*, *Kdm2a, Ikzf1, Klf3)*, enhanced *JAK/STAT5* signaling (*Jak1*) and modification of the *CDKN2A* tumor-suppressor pathway (*Bmi1*, *Cdkn2a*).

The exomes of CLL samples average less than one coding driver mutation per sample, making mechanisms of transformation difficult to identify. The Eµ-*TCL1* transgenic model develops a disease that is phenotypically similar to CLL. The B cells of these mice were mutagenized using a conditional SB transposase switched by *CD19*-Cre (pan B cell) (44, 59). Transposon mobilization decreased latency of Eµ-*TCL* leukemias and integrations of 15 mice yielded 8 CIS loci, four of which implicate Nf-kappaB signaling (*Nfkb1*, *Tab2*, *Map3K14*, and *Nfkbid*). NF-kappaB activating mutations are rare in the coding regions of human CLL however it has been shown that CLL cells are frequently dependent on extracellular signaling pathways that activate Nf-kappaB (77) and several mouse models of CLL/SLL have constitutive activation of NF-kappaB pathways (78–80).

The Cre strains used to switch conditional SB alleles need not be entirely B cell specific in order to generate B cell malignancies. The *Cnp*-Cre strain expresses Cre in the nervous system and splenic germinal centers (47). Combining *Cnp*-Cre with an SB11 conditional allele, a transposon concatemer and a conditional oncogenic point mutation of tumor suppressor *Trp53* (*Trp53* ^R270H^) generated a mixture of solid tumors as well as lymphoid malignancies with an FL/DLBCL like phenotype. Integrations from 23 samples identified *Bach2* as the most frequently mutated locus. *Pten* disrupting mutations were also identified and the authors verified *Pten* as a tumor suppressor in the *Cnp*-Cre driven model. These results are consistent with human germinal center B cell DLBCL where *PTEN* is mutated or deleted (81) and reduced expression correlates with AKT activation (82).

More recently the piggyBac transposon has also been adapted for somatic insertional mutagenesis. Mice expressing constitutive piggyBac transposase were crossed with a gene trap piggyBac transposon concatemer allele onto a *Blm^m3/m3^* hypomorph background (48). Gene trap transposons are more likely to generate intragenic loss of function mutations which are in turn more likely to be selected if they occur in tumor suppressors. The use of a *Blm* hypomorph background to enhance loss of heterozygosity events is the same strategy used to enrich for tumor suppressors in the retroviral screen of AKXD *Blm^m3/m3^* mice cited in the previous section (40). A spectrum of solid and hematological tumors were generated, with the majority of mice having DLBCL like B lymphoid malignancies. These had recurrent amplification of *Bcl11a* and *Rel*, this region also being amplified in human DLBCL. Integration site cloning using the semiquantitative QiSeq insert cloning protocol (83) on 43 samples identified nearly 300,000 integrations. Inactivating/disrupting mutations were observed in tumor suppressors known to be mutated in human DLBCL and there was significant overlap between the orthologs of CIS genes and the set of genes that are down regulated in human DLBCL *vs* non-malignant B cells. This suggests CIS genes may inform the identification of genes that are down regulated in human DLBCL by epigenetic variation and/or large-scale deletions.

### Quantitative Analyses of Subclonal Integration Mutations

Improvement in next generation sequencing platforms and ligation mediated PCR methods have increased the throughput and sensitivity of integration site cloning. Ligation mediated-PCR methods such as vectorette and splinkerette-PCR use a non-complimentary adaptor that facilitates enrichment of integrated sequences separately from the remainder of the genome (84–86). Shearing of tumor DNA prior to construction of sequencing libraries allows the abundance of each integration within a sample to be quantitated by counting the number of unique read fragment lengths mapping to each integration (83, 87–89). This approach to quantitation minimizes sequence and amplification biases particularly when compared to libraries prepared by non-random digestion at restriction enzymes recognition sites. Other studies have quantitated integrations through analyses of whole genome amplification of single cells (SBCapSeq) (90, 91) or using unique molecular identifiers (LUMI-PCR) (92).

Collectively, these recent methods allow affordable, genome wide identification of thousands of subclonal mutations with a sensitivity spanning more than two orders of magnitude. This quantitative coverage in turn allows estimation of the order in which lesions occurred (93) and cloning large numbers of subclonal mutations provides the statistical power needed to derive genetic associations between early-stage clonal mutations and late-stage subclonal mutations (29). Translating these types of analyses to human tumors is currently limited by depth of sequencing coverage. Currently whole genome sequencing of human tumors rarely exceeds 100x coverage and therefore offers less sensitive detection of subclonal mutations than recent integration site cloning studies. Nonetheless, recent studies of single cell sequencing of human tumors include thousands of cells which can therefore identify subclonal mutations within limited/targeted regions of the genome (94–97).

The scale of these datasets requires innovations in statistical methodology, both to identify selected mutations and to demonstrate associations between them. For density based estimates of mutation selection (98–100) the background integration biases of insertional mutagens can be compensated for by the use of unselected cell populations for comparison with tumor mutation distributions (29, 93, 101). Alternatively, identifying local ratios of forward/reverse strand mutations throughout the genome is analogous to using ratios of synonymous and non-synonymous coding mutations in that it does not require correction for integration site biases of the mutagen (29, 93). Strand bias analyses requires a higher density of integrations to reach statistical significance and will only identify loci with a strong bias in one direction or the other.

Analyses of concurrent mutations is complicated by the inclusion of subclonal mutations. When sequencing coverage is deep enough to include very subclonal mutations, some of the most frequently mutated loci can be found mutated in 100% of samples. Any two loci found to have subclonal mutations in all samples will appear to be 100% concomitant, even when these mutations are not found within the same subclones of each sample. For this reason, quantifying the significance of concurrent mutations requires the use of contingency table tests that exclusively use clonal mutations i.e. mutations with a high likelihood of occurring within the same cells. Alternatively it is possible to use asymmetrical contingency tables that classify all tumors on the basis of their early stage “trunk” clonal mutations and/or germline mutations at one locus and then test for the distribution of all mutations (both clonal and subclonal) at other loci (29, 102, 103). The number of genome-wide pairwise tests that can be performed on these cohorts can number in the thousands and this in turn requires stringent multiple testing correction to limit false positive associations. Nonetheless, with high coverage of subclonal mutations it is now possible to identify hundreds of significantly co-mutated loci pairs from a cohort of several hundred mice infected with MoMuLV (29).

### Forward Genetic Screens by Exome Sequencing

The expense of exome and whole genome sequencing is generally reserved for the study of human tumors however, an increasing number of mouse tumor exomes have also been sequenced, albeit with cohort sizes that are far smaller than their human counterparts. In most cases this sequencing is to verify that the model accurately recapitulates human disease, however, some studies also suggest or verify candidate drivers where coding mutations in human cohorts fail to reach statistical significance. Several examples are discussed below.

Combining Cre recombinase inducible alleles of mutant *Myd88* (L252P) and *Rosa-26* expressed *Bcl2* with germinal center specific *Cγ1-Cre* generated a human ABC-DLBCL like model (60). Sequencing samples from 17 mice confirmed mutation of human DLBCL drivers including *Pim1*, *Myc*, *Kmt2d*, *Nfkbia*, *Stat3*, *Pou2f2*, and *Hist1h1e*. In a similar study, conditional expression in germinal center B cells of *Myc* and constitutively activated *Akt* (fusing p85 to p110) gave rise to Burkitt’s lymphoma (BL) like disease with recurrent mutations in *Ccnd3*, which is mutated in BL and known to regulate germinal center B cell proliferation (49).

*TET1* and *TET2* encode methylcytosine dioxygenase enzymes that are thought to play a role in demethylation of DNA by catalyzing conversion of 5-methylcytosine to 5-hydroxymethylcytosine. *TET2* is mutated in a range of hematopoietic malignancies including a subset of DLBCL where mutations can be traced back to HSC populations, suggesting *TET2* mutation is an early event in lymphomagenesis (104). Mice lacking *Tet2* globally or in B cell subpopulations (*CD19*-Cre or *Vav*-Cre) develop B cell lymphoma (57, 105). One study sequencing six B cell tumors matched to germline counterparts revealed thirty-four acquired mutations, albeit not recurrently mutated, presumably due to the limited cohort size. Nonetheless, more than half of these genes are also mutated in either DLBCL, CLL or both.

*TET1* is not mutated in human B malignancies however it is downregulated in human DLBCL and FL which inconclusively suggests a role for *TET1* in human B lymphoma. *Tet1* deficient mice develop mature B cell malignancies which when sequenced were found to have missense mutations in the mouse homologues of known human B cell malignancy drivers including *Gna13*, *Kmt2d*, *Myd88, Cd83*, *Pim1, Cd79B* and *Fas* (106). Additional mutations were observed in linker histone genes, histone variants and histone modifying enzymes. Histone linker mutations are a feature of FL (107). These mutations corroborate a role for *TET1* down regulation in human B cell malignancies.

Mouse models that accurately recapitulate human disease can also identify novel drivers. The Eμ *-Myc* model is a mainstay of B cell leukemia/lymphoma research but exomes from this model have only recently been sequenced (54). Sequencing of 23 lymphoma exomes revealed recurrent mutations in known drivers such as *Trp53*, *Cdkn2a* and *Kras*, but also the “*BCL6* corepressor” gene *Bcor*. Inactivating mutations in *Bcor* accelerated Eμ*-Myc* driven lymphomas in transplantation assays. An independent study sequenced *NUP98/PHF23* transgenic mice which primarily develop T cell lymphoma but in a minority of cases also develop progenitor B-1 type ALL. This study also identified *Bcor* mutations in addition to mutations of *Jak1*, *Jak2*, *Jak3* and *Stat5a* (51, 56). *Bcor* loss of function mutations were subsequently found to cause B-1 progenitor ALL in mice (108).

Aside from driver detection, exome sequencing can also be a powerful tool to examine the mutagenic processes of early premalignant states under defined experimental conditions. Sungalee *et al.* developed an innovative model of t(14;18) translocation where human *BCL2* expression from an Eμ enhancer is sporadically induced by RAG recombinase in pro/pre B cells (50). Repeat immunization demonstrated a survival advantage for *hBCL2* expressing cells after multiple rounds of germinal center reentry. Exome sequencing was performed on *BCL2*-enriched germinal center and memory cell fractions from three chronically immunized mice and a control (empty vector) mouse. B cell subsets of the *BCL2*-transduced mice had between 111 and 2,565 nonsilent SNVs compared with 63 to 70 SNVs in the control mouse, despite the absence of overt morphological differences between *hBCL2* and control animals. This model demonstrates a role for *BCL2* in enhanced survival of cells that accumulate potentially oncogenic off target mutations of AID.

### *Pax5* Driven Models of B ALL; A Case Study of Multimodal Screens Converging on Common Findings

*PAX5* encodes a B cell development transcription factor that activates B cell specific genes and represses genes of alternative lineages. It is one of the most frequently altered genes in B ALL and a varied spectrum of somatic rearrangements, translocations, mutations and deletions have been observed at this locus (76, 109–111). Karyotypic analysis indicates translocations are early events whereas deletion of a second allele appears to be a secondary later event. Germline point mutations that inhibit *PAX5* function also increase the likelihood of B ALL and some are accompanied by somatic deletion of the other *PAX5* allele or cooperating *PAX5* mutations (112–115). The discovery of frequent *PAX5* deletion and translocations in human ALL was rapidly followed by a large number of studies using some form of *Pax5* deletion or translocation in mice to model B ALL. To date six independent studies have included exome sequencing and/or insertional mutagenesis of mouse B ALL samples generated by mutation of *Pax5*. The convergence of findings of these studies demonstrates the value of multimodal screening in mice to validate mutation profiles of human tumors.

*Pax5* heterozygosity combined with thymectomy skews ENU and MoMuLV driven lymphomas toward a B lineage disease since thymectomy removes competing T cell malignancies (28). Array-CGH copy number analyses of these malignancies identified amplification of chromosome 15 (and consequently *Myc*) and focal deletions of the second allele of *Pax5*. Exome sequencing revealed B ALL samples from ENU treated mice had 5-fold more exonic mutations than MuLV infected mice and identified recurrent mutations in the human ALL mutated genes: *Pax5*, *Jak3*, *Ptpn11*, *Jak1*, and *Nras*. *Pax5* mutations were almost exclusively within the DNA-binding paired domain and equivalent to mutations observed in human B ALL. *Jak1* and *Jak3* mutations were located in the pseudokinase domains of *JAK1* and *JAK3*, and are known to induce cytokine-independent proliferation and activation of downstream signaling pathways sensitive to JAK inhibitors. MoMuLV CIS included an overlapping set of B ALL drivers including *Myc*, *Stat5b*, *Zeb2*, *Jak1*, *Jak3*, *Ikzf1*, *Gsdmc*, *Ebf1* and *Ptpn11*.

In another study multiple Cre strains (*Cd19*, *Mb1(Cd79a)* or *Mx1*) were used to induce conditional expression of a *E2A/PBX1* fusion, resulting in B cell precursor ALLs, which arrested at the pro B/large pre B II stages in a manner similar to human *E2A/PBX1* ALL (53). Copy number analyses and exome sequencing of leukemia samples revealed that 30% harbored inactivating mutations or deletion of *Pax5* that resulted in decreased expression. Combining *E2A/PBX1* with *Pax5* deletion increased the penetrance and shortened the latency of leukemia. Other mutated loci from *E2A/PBX1* tumors included *Cdkn2a*, *Jak1*, *Jak3*, *Ptpn11* and *Kras*.

The *ETV6/RUNX1*-SB model described in the previous section (43) was subsequently combined with heterozygous deletion of *Pax5* which increased the incidence of B-cell precursor (BCP)-ALL (45). Targeted exome sequencing identified recurrent mutations in *Jak3*, *Trp53* and *Jak1*, with *Jak1/3* mutations again being found in the pseudokinase domain. Insertional mutagenesis identified 6 CIS, four of which (*Zfp423, Cblb, Stat5b and Foxp1*) have well-characterized roles in B cell maturation. Increased *ZNF423* expression is associated with *ETV6/RUNX1*+ B ALL and *Pax5* or *Ebf1* mutations synergize with *STAT5* in B ALL. It is worth noting this mutation profile is more likely a function of *Pax5* loss since a different model of *ETV6/RUNX1* with wild type *Pax5* primarily identified loss of function mutations in the KDM lysine demethylase family (55).

B ALL models have also been used to study the relationship between gut microbiota and leukemia incidence (52, 55, 61). *Pax5* heterozygous mice do not develop leukemia when housed in specific pathogen free (SPF) conditions however they are prone to precursor B ALL when exposed to common pathogens and sequencing of these leukemias identified recurrent *Jak3* mutations (52). In an independent study it was also shown that *Pax5* heterozygous mice or mice bearing a *Sca1-ETV6/RUNX1* transgene also do not develop B ALL when housed in SPF conditions. Nonetheless both mouse strains had sufficiently altered immune responses to alter the gut microbiome composition when housed in a non-SPF facility and *Pax5* heterozygous mice in SPF conditions treated with antibiotics developed B ALL (61). The authors hypothesize that dysbiosis caused by non-SPF housing or the absence a normal gut biome in SPF conditions with antibiotics drives disease on this background, however secondary mutations were also required. Exome sequencing of 17 B ALL cases from non-SPF conditions identified recurrent mutations affecting the JAK/STAT and RAS signaling pathways (*Jak1*, *Jak3*, and *Ptpn11*). Non-SPF mice treated with antibiotics also harbored recurrent mutations in *Kit*, *Flt3*, and *Cbl* that have not been observed in other *Pax5* ^+/–^ cohorts. This may suggest that selection of secondary mutations reflects differences in gut biome.

PAX5 is frequently mutated by translocation events creating fusion proteins in human tumors and some of these have been shown to cause B ALL in mice (58, 116). In one of these studies exome sequencing of five leukemic mice expressing a *PAX5/ENL* fusion identified mutations in *Ptpn11*, *Kras*, *Pax5*, and *Jak3* genes (58). Of these genes *PAX5, PTPN11*, *KRAS* and its homologue *NRAS* were found recurrently mutated across a panel of human B ALL samples from diverse subtypes.

These six independent studies using complementary experimental designs all identify mutations in *Jak3* by exome sequencing and in some cases also by insertional mutagenesis. Furthermore, four studies identified mutations in *Jak1*, three studies identified Ras family member mutations (albeit through different mechanisms) and four had *Ptpn11* mutations. Collectively this work demonstrates how mutagenesis and exome sequencing of mouse models with defined lesions can reliably and reproducibly reveal cooperating mutations in independent cohorts, and that *Pax5* heterozygosity in mice faithfully reproduces mutational profiles observed in human B ALL.

## Reverse Genetic Models

Over the past decade the expanding number of candidate mutations associated with B cell malignancies has increased the requirement for medium to high throughput methods of validation. There is an extensive literature of transgenes, knockouts and conditional alleles for B cell lymphomas [reviewed in (117–121)]. Although tissue specific switching of conditional alleles is arguably the gold standard for mouse tumor models, the obvious drawback of this approach is the time taken to generate strains and cohorts of multiallelic models. In this section we discuss higher throughput reverse genetic models including transplantation/adoptive transfer models with an emphasis on models using primary cells transduced with viral constructs. We also summarize recently developed methods for rapid generation of multiallelic strains and several approaches to temporal control of gene expression.

### B Cell Malignancy Models From Transplantation of Virus Transduced Cells

Early transplantation-based B cell malignancy models used cell lines derived from spontaneous malignancies injected into syngeneic hosts and/or human malignant cell lines injected as xenografts into immunocompromised mouse strains [reviewed in (122)]. More recently primary B cell malignancies have been transduced with viral constructs prior to transplantation, either to overexpress oncogenes (123–125) or to express shRNAs/sgRNAs that validate essential genes, therapeutic targets and tumor suppressors (126–134) (**Table 3**).

**Table 3:** Reverse genetic B cell malignancy transplantation assays using primary lymphomas.

Publication	Donor lymphoma genotype	Recepient	Donor cell type	Genes delivered	Malignancy type
Schmitt CA et al. (123)	Eμ-Myc	syngeneic	primary B lymphoma	*Bcl2* ORF virus	B lymphoma
Schmitt CA et al. (124)	Eμ-Myc	syngeneic	primary B lymphoma	*Bcl2* ORF virus	B lymphoma
Schmitt CA et al. (125)	Eμ-Myc, Eμ-Myc;Trp53^+/-^, Eμ-Myc;p19^ARF+/-^ & Eμ-Myc;Cdkn2a/p19 ^ARF+/-^	syngeneic	primary B lymphoma & fetal liver derived HSPCs	*Bcl2*, Casp9 dominant negative *Cdkn2a* & *p19ARF* ORF viruses	B lymphoma
Schmitt CA et al. (135)	Eμ-Myc or Eμ-Myc;Trp53^+/-^	syngeneic	primary B lymphoma & fetal liver derived HSPCs	*Bcl2* & *Casp9* dominant negative ORF viruses	B lymphoma
Refaeli Y et al. (126)	Eμ-Myc;BCR^HEL^ & Eμ-Myc;BCR^HEL^;sHEL	syngeneic	primary B lymphoma	Cd79a (lgα) & Cd79b (lgβ) shRNA virus	B lymphoma
Young RM et al. (127)	Eμ-Myc;BCR^HEL^ & Eμ-Myc;BCR^HEL^;sHEL	syngeneic	primary B lymphoma	*Syk* shRNA virus	B lymphoma
Meacham CE et al. (136)	Eμ-Myc;p19^ARF-/-^	syngeneic	primary B lymphoma	1000 gene shRNA virus library	B lymphoma
Mu P et al. (137)	Eμ-Myc;miR-17~92*^ff/ff^*;Rosa26-Cre-ER	athymic nude mice	primary B lymphoma	*Pten* shRNA & *miR-17~92* viruses	B lymphoma
Zuber J et al. (128)	Eμ-Myc;Trp53^-/-^	syngeneic	primary B lymphoma	*Rpa3* shRNA viruses	B lymphoma
Malina A et al (138)	Eμ-Myc;p19^ARF-/-^	syngeneic	primary B lymphoma	*Trp53* & *Rosa26* sgRNA viruses	B lymphoma
Cao Z et al. (129)	Eμ-Myc, B6RV2 leukemia cells, primary human Burkitt's lymphoma	syngeneic or NSG	primary B lymphoma or B leukemia cell line	*Notch1 Notch2 Hey1* & *Jag1* shRNA viruses	B lymphoma
Hoellein A et al. (130)	Eμ-Myc	syngeneic	primary B lymphoma	*Sae2* shRNA viruses	B lymphoma
Matthews GM et al. (131)	Eμ-Myc & Eμ-Myc;Hdac1^-/-^	syngeneic	primary B lymphoma	*Hdac2* & *Hdac3* shRNA viruses	B lymphoma
Duque-Afonso et al. (132)	E2A-PBX1;CD19.Cre & E2A-PBX1;Mx1.Cre	syngeneic	primary B ALL	*Plcg2* shRNA virus	B ALL
Braun CJ et al. (134)	Eμ-Myc;p19^ARF-/-^ & Bcr-Abl	syngeneic	primary B lymphoma	*Trp53*, *Chek2* & 25 gene library sgRNAs & dCas9/dCas9-VP64 viruses	B lymphoma & B ALL
Li X et al. (133)	Eμ-Myc;p19^ARF-/-^	syngeneic	primary B lymphoma	*Utx* shRNA & *Efnb1* ORF virus	B lymphoma

Primary B lymphomas have also been used to perform *in vivo* shRNA screens. In one study shRNAs against 1000 candidate cancer genes were selected to identify genes that are required for B lymphoma growth *in vivo* and this identified regulators of actin dynamics and cell motility (136). In a more targeted study, combining an *in vitro* screen with *in vivo* verification, conditional deletion of the *miR-17∼92* cluster was found to cause apoptosis in Eμ-*Myc* lymphomas. *In vitro* screening of shRNAs against targets of these microRNAs identified *Pten* suppression as a mediator of *miR-17∼92* survival signals which was verified when shRNAs against *Pten* rescued *in vivo* lymphoma growth of *miR-17∼92* deleted Eμ-*Myc* lymphoma cells (137).

Studying the transition of non-malignant cells into lymphomas and leukemias requires the use of untransformed primary cells. Retroviral transduction of primary cell suspensions from spleen, bone marrow and fetal liver can reveal the transforming potential of candidate genes and mutations. In early experiments both viral and cellular genes were found to facilitate colony formation in agar, clonal outgrowth of cell lines and development of malignancies from adoptive transfer into irradiated or immuno-compromised recipients [reviewed in (139, 140)]. The malignancy subtypes generated are a function of the genes being transduced and the lineage of infected cells, with B cell malignancies being successfully generated from wild type donor cells by using cellular genes (141, 142), acute transforming retrovirus oncogenes (143–147) and the fusion transcripts of oncogenic translocations (145, 148–152) (summarized in **Table 4**). Experiments using the mixed populations of cell types from whole bone marrow as donor cells can yield a mixture of malignancy subtypes from a single cohort. The use of purified target cells offers greater control of malignancy subtype, and protocols for the culture and transduction of purified HSCs, hematopoietic stem and precursor cells (HSPCs), B lineage precursors/progenitors and splenic B cells (188–190) have all been adapted to generate B lineage malignancies.

**Table 4:** Reverse genetic B cell malignancy transplantation assays using primary cells.

Publication	Donor primary cell genotype	Recipient	Donor cell type	Genes delivered	Malignancy type
Schwartz et al. (143)	wild type	syngeneic	bone marrow derived pre B cells & sIg+ B cells	v-*myc* & v-*H-ras* ORF viruses	B lymphoma
McLaughlin et al. (148)	wild type	syngeneic	bone marrow derived immature B cells	*BCR-ABL1* p210 ORF virus	B lymphoma
Heard et al. (144)	wild type	syngeneic	bone marrow	v-*fms* ORF virus	B cell lymphoma & erythroleukemia
Alexander et al. (153)	Eμ-*Myc*	nude mice	bone marrow	v-*H-ras* & v-*raf* ORF virus	B lineage subcutaneous tumors
Keliher et al. (145)	wild type	syngeneic	bone marrow	Abelson MuLV (v-*abl*) & *BCR-ABL1* p210 ORF virus	pre B cell lymphoma & myeloproliferative disease
Daley et al. (149)	wild type	syngeneic	bone marrow	*BCR-ABL1* p210 ORF virus	CML, B/T ALL & macrophage tumors
Elefanty et al. (150)	wild type	syngeneic	bone marrow	*BCR-ABL1* ORF virus	pre-B lymphoid, T lymphoid, macrophage, erythroid & mast cell tumors
Hawley et al. (146)	wild type	syngeneic	bone marrow	v-*H-Ras* ORF virus	pre-T-cell thymic lymphomas & pre-B-cell lymphoblastic leukemia/lymphomas
Kitayama et al. (141)	wild type (*Kit* ^+/+^ )	*Kit* ^W/W-v^	bone marrow	*Kit^G599^* & *Kit^V814^* ORF viruses	B cell leukemia
Thome et al. (147)	wild type	syngeneic	bone marrow derived pre B cells	Abelson MuLV (v-*abl*)	B lymphoma
Kuefer et al. (151)	wild type	syngeneic	bone marrow	*NPM-ALK* fusion ORF virus	B lineage large-cell lymphoma
Hawley et al. (142)	wild type	syngeneic	bone marrow (5-FU-treated)	*Flt3* ORF virus	B cell and/or myeloid hematologic malignancies
Li et al. (152)	wild type	syngeneic	bone marrow (5-FU treated & untreated)	*BCR-ABL1* p190, p210 & p230 ORF viruses	CML, B ALL & macrophage tumors
Sexl et al. (154)	*wild type & Stat5a/b* ^DeltaN/DeltaN^	syngeneic	bone marrow	*BCR-ABL1*p210 & p185 ORF viruses	B cell & myeloid leukemia
Schmitt et al. (125)	Eμ-*Myc*, Eμ-*Myc*;*Trp53* ^+/-^, Eμ-*Myc*;*p19* ^ARF+/-^ & Eμ-*Myc*;*Cdkn2a/p19* ^ARF+/-^	syngeneic	primary B lymphoma & fetal liver derived HSPCs	*Bcl2*, Casp9 dominant negative *Cdkn2a* & *p19ARF* ORF viruses	B lymphoma
Schmitt et al. (135)	Eμ-*Myc* & Eμ-*Myc*;*Trp53* ^+/-^	syngeneic	primary B lymphoma & fetal liver derived HSPCs	*Bcl2 & Casp9* dominant negative ORF viruses	B lymphoma
Hemann et al. (155)	Eμ-*Myc*	syngeneic	fetal liver derived HSCs	*Trp53* shRNAs	B lymphoma
Hemann et al. (156)	Eμ-*Myc* & wild type	syngeneic	fetal liver derived HSCs	*Puma/Bbc3* shRNA virus	B lymphoma
Wendel et al. (157)	Eμ-*Myc*, Eμ-*Myc*;*p19* ^ARF+/-^ & Eμ-*Myc*;*Trp53* ^+/-^	syngeneic	fetal liver derived HSCs & primary B lymphoma	*Bcl2*, *Eif4e*, constitutive *Akt* & dominant negative *Casp9* ORF viruses	B lymphoma
Hu et al. (158)	wild type & *Lyn* ^-/-^;*Hck* ^-/-^;*Fgr* ^-/-^	syngeneic	bone marrow (5-FU-treated & untreated)	*BCR-ABL1* p210 ORF virus	CML & B ALL
Hemann et al. (159)	wild type, *Bim* ^-/-^, *Trp53* ^+/-^ & *Trp53* ^-/-^	syngeneic	fetal liver derived HSCs	*Myc* & *Myc* mutant ORF viruses	B lymphoma
He et al. (160)	Eμ-*Myc*	syngeneic	fetal liver derived HSCs	*mir-17–19b* microRNA virus	B lymphoma
Herbst et al. (161)	*Trp53* ^+/-^	syngeneic	fetal liver derived HSCs	*Myc* & *Myc* mutant ORF viruses	B lymphoma
Williams et al. (162)	wild type, *p19* ^ARF+/−^ & *p19* ^ARF−/−^	syngeneic	bone marrow derived pre B cells	*BCR-ABL1*p210 & p185 ORF viruses	B lympholeukemia
Hoelbl et al. (163)	wild type & *Stat5a/b* ^-/-^	*Rag2* ^-/-^	fetal liver cells & bone marrow	*BCR-ABL1* p185 ORF virus & Abelson MuLV (v-*abl*)	B lymphoid leukemia
Barabé et al. (164)	wild type (human)	NSG & B-NOD/SCID	human umbilical cord blood stem and progenitor cells (Lin– CB)	*MLL-ENL* (*KMT2A-MLLT1*) & *MLL-AF9* (*KMT2A-MLLT3*) ORF viruses	B precursor acute lymphomblastic leukemia & AML
Wang etal (165)	wild type, *Cdkn2a/p19* ^ARF+/-^ & *p19* ^ARF−/−^	syngeneic	bone marrow HSCs, common lymphoid progenitors, pro/pre B cells	*BCR-ABL1*p210 ORF virus	B ALL & CML
Bric et al. (166)	Eμ-*Myc*	syngeneic	fetal liver derived HSPCs	1000 gene shRNA virus library	B lymphoma
Hoelbl et al. (167)	*Stat5^fl/fl^* & *Mx1Cre^+^;Stat5^fl/fl^*	*Rag2^-/-^γc^-/-^*	bone marrow (5-FU treated & untreated)	*BCR-ABL1* p210 ORF virus & Abelson MuLV (v-abl)	B lymphoid leukemia & CML
Nakagawa et al. (168)	wild type	SCID	fetal liver derived pro B cells	*Bcl2*, *Myc* & *Ccnd1* ORF viruses & human ORF virus library	immature B cell lymphoma
Bouquet et al. (169)	wild type	*Rag1* ^-/-^	fetal liver derived pre B cell lines	*Myc* & *Pim1* ORF viruses	pre B cell hyperplasia
Kovacic et al. (170)	wild type, *Mx1Cre* ^+^;*Stat5* ^+/+^ & Mx1Cre^+^;*Stat5* ^fl/fl^	syngeneic	bone marrow, long‐term HSCs, lymphoid‐myeloid progenitors & HSC‐depleted marrow	*BCR-ABL1*p210 & p185 ORF viruses	B acute lymphomblastic leukemia & CML
Leskov et al. (171)	wild type (human)	NSG	human umbilical cord blood derived CD133+ HSCs	*MYC* & *BCL2* ORF viruses	pre B cell lymphoma/leukemia
Arita et al. (172)	wild type	NSG	spleen derived induced germinal centre B cells	*Bcl2l1 (Bcl-xl)* & *Myc* ORF viruses	mature B cell lymphoma
Aubrey et al. (173)	Eμ-*Myc*	syngeneic	fetal liver derived HSPCs	*Trp53* sgRNA virus	B lymphoma
Ortega-Molina et al. (174)	VavP-*BCL2*	syngeneic	fetal liver derived HSCs	*Kmt2d* shRNA virus	B lymphoma
Scheicher et al. (175)	wild type & *Cdk6* ^-/-^	NSG	bone marrow	*BCR-ABL1*p210 ORF virus	B leukemia
Jiang et al. (176)	VavP-*BCL2*	syngeneic	fetal liver derived HSCs	*Crebbp* shRNA virus	B lymphoma
Katigbak et al. (177)	Eµ-*Myc*	syngeneic	fetal liver derived HSPCs	sgRNA virus library	B lymphoma
Wolf et al. (178)	*Myc*-GFP	*Rag1* ^-/-^	fetal liver derived pre B cell line	ORF virus library	B lymphoma
Wolf et al. (179)	GFP-rtTA	*Rag1* ^-/-^	fetal liver pre BI cells	*Myc* & *Bcl-xl* ORF viruses	plasmablast/plasma cell hyperplasia
van Oosterwijk et al. (180)	*wild type, p19*^ARF−/−^ , *p15* ^smARF+/+^ & *p15* ^smArf−/−^	syngeneic	bone marrow derived pre B cells	*BCR-ABL1* p185 ORF	B ALL
McHugh et al. (181)	wild type (human)	NSG	human fetal liver derived derived CD34+ hematopoietic progenitor cells	EBV & KSHV infection	B lymphoma
Reimer et al. (182)	wild type (human)	NSG	human cord blood derived HSPCs	*MLL*(*KMT2A*) & *ENL*(*MLLT1*) sgRNA viruses	B ALL & MLL
Lefebure et al. (54)	Eµ-*Myc*	syngeneic	fetal liver cells	*Trp53* & *Bcor* shRNA/sgRNA viruses	B lymphoma
Katigbak et al. (183)	Eµ-*Myc*;*Rosa26*-rtTA;*Col1A1*-TRE-Cas9-IRES-GFP/CAG-rtTA	syngeneic	fetal liver derived HSPCs	*Trp53* sgRNA virus	B lymphoma
Janic et al. (184)	Eμ-*Myc*, Eμ-*Myc;Bbc3^-/-^* & *Bbc3^-/-^;Cdkn1a^-/-^*	syngeneic	fetal liver derived HSPCs	*Trp53* sgRNA viruses & shRNA library viruses	B lymphoma
Jeong et al. (185)	wild type (human)	NSG	human cord blood derived HSPCs	*AF9*(*MLLT3*) & *MLL*(*KMT2A*) sgRNAs	B ALL, AML, or MPAL
Yin et al. (108)	*NUP98-PHF23*	syngeneic	fetal liver cells & bone marrow	*Bcor* shRNA/sgRNA viruses	progeniotor B1 acute lymphomblastic leukemia
Weber et al. (48)	Eµ-*Myc*;*Rosa26*-Cas9	syngeneic	fetal liver derived HSPCs	*Rfx7* & *Phip* sgRNA viruses	B lymphoma
Rajan et al. (186)	wild type	syngeneic	fetal liver derived HSCs	*Npm1* & *Alk* sgRNA plasmids	T & B anaplastic large cell lymphoma
Verma et al. (187)	wild type	syngeneic & *Mmp9* ^-/-^	bone marrow	*BCR-ABL1* ORF & *TNF* shRNA viruses	B acute lymphomblastic leukemia

Combining both sensitized germline alleles with transduction of retroviruses also influences the cell type and developmental stage of the disease being modelled. One of the most frequently used strain to derive B cell malignancies by adoptive transfer of transduced cells is the Eμ-*Myc* model of Burkitt lymphoma (191). When bone marrow derived pre B cells from Eμ-*Myc* transgenic mice were transformed by transduction with v-*H-ras* or v-*raf*, the resulting clones forming subcutaneous tumors in nude mice (153). Later protocols using transplanted Eμ-*Myc* fetal liver cells (typically HSCs and HSPCs) yielded B cell lymphomas that could be accelerated by loss of tumor suppressors in the germline (e.g. *Trp53*, *Cdkn2a, p19^ARF^*) and by retroviral expression of cooperating ORFs (125, 135, 157, 162). The use of fetal liver cells is particularly useful for the generation of lymphomas where one or more germline alleles prevent normal development after E17. This approach has been used to demonstrate that deletion of the essential mediators of apoptosis *Apaf1* and *Casp9* does not affect reconstitution by fetal liver HSCs (192) or alter the phenotype or latency of lymphomas derived from reconstitution by Eμ-*Myc* fetal liver HSCs (193).

Viral constructs capable of robust expression of short RNAs have facilitated rapid verification of putative tumor suppressors and oncogenic microRNAs. In an early study a series of *Trp53* shRNAs introduced into Eμ-*Myc* HSCs were found to accelerate lymphomagenesis in a manner that correlated with the strength of knockdown (155) and similar results were obtained for the *mir-17–19b* microRNA (160) and for shRNAs against *Puma* (*Bbc3*, a proapoptotic downstream target of *Trp53) (* 156) and *Bcor* (a tumor suppressor of Burkitt lymphoma) (54). The Eμ-*Myc* model has also been used to screen pools of shRNAs against a set of 1000 known and putative cancer genes for tumor suppressor activity. Infecting fetal liver HSCs and transplanting them into recipients identified *Sfrp1*, *Numb*, *Mek1*, *Ang2* as tumor suppressors, as well as identifying known components of the DNA damage response machinery including *Rad17 (* 166). A more focused shRNA screen of known targets of *Trp53* was conducted using fetal liver HSCs of mice that were either Eμ-*Myc Puma*-/- or *Cdkn1a*(*p21)*-/-*Puma*-/- in order to identify targets of p53 that were responsible for its tumor suppressor role independently of *Cdkn1a* or *Puma* (184). This identified a spectrum of shRNAs against DNA damage repair genes including *Mlh1*, *Msh2*, *Rnf144b*, *Cav1* and *Ddit4* which were capable of accelerating Eμ-*Myc* fetal liver HSC transplant lymphomas.

The most common strains used in these transplantation experiments are wild type mice, Eμ-*Myc* transgenics, *Trp53* mutants and *Cdkn2a*/*p19^ARF^* mutants however many other strains have also been used to probe the cooperative or epistatic relationships of transduced genes with germline alleles (108, 158, 159, 161, 162, 165, 174–176, 180, 187) (summarized in **Table 4**). HSCs transduced with *Myc* retroviruses develop into aggressive pre B cell lymphomas when transplanted (159), and point mutants of *Myc* typically found in Burkitt’s lymphoma increased penetrance and accelerated latency. Replacing wild type donor cells with *Trp53^-/+^*, *Trp53^-/-^* or *Bim^-/-^* donor HSCs accelerates latency of wild type *Myc* more than *Myc* mutants, suggesting these mutations reduce *Myc* induced apoptosis mediated by *Trp53*/*Bim* and reduce selection for *Trp53* inactivation. Thus, epistatic effects and mutation redundancy can be rapidly investigated using germline alleles that predispose animals to disease.

### Modelling the Genetics and Cell of Origin of *BCR/ABL* Leukemias

The “Philadelphia chromosome” translocation, t(9;22)(q34;q11), that expresses the *BCR/ABL1* translocation product is observed in both human CML and B ALL. Retroviral transduction of hematopoietic cells with the *BCR/ABL1* fusion transcript and transplantation into syngeneic recipients is one of the most extensively studied transplantation models in the literature. *BCR/ABL1* transduced bone marrow gives rise to various lineages of malignancies including, B lineage, myeloid and mixed lineage leukemias. Disease subtype and latency can be altered by varying donor cell lineage, genotype, culture protocols and the use of different *BCR/ABL1* isoforms. B lineage disease is particularly promoted by culture conditions that enhance pro/pre B differentiation and the use of the p185(p190) isoform (152, 170). Myeloid disease can be promoted by the p210 isoform and by pre-treating donor mice with 5-fluorouracil which depletes bone marrow of cycling cells and prompts cycling of quiescent HSCs (152, 170).

Mutations that determine which cell lineages can form different malignancy subtypes have been extensively studied using this model. Transplantation of purified HSCs transduced with *BCR/ABL1* retrovirus typically gives rise to CML, however loss of both *Cdkn2a/p19^ARF^* or only *p19^ARF^* in donor cells gives rise to B ALL (165). This is consistent with the observation of concomitant deletion of the *CDKN2A/p14^ARF^* locus in human B ALL but not in CML. *p19^ARF^* loss also enables B ALL to develop rapidly from *BCR/ABL1* transduced common lymphoid progenitors, pro B cells and pre B cells. Furthermore, *p19^ARF^* null pro B cell derived disease was phenotypically different from wild type pro B cell derived disease which had substantially lower penetrance and delayed kinetics. When compared to *p19^ARF^* null pro B cell derived disease, *p19^ARF^* null HSC derived B ALL had greater colony forming potential in methylcellulose, greater resistance to dexamethasone and reduced resistance to the kinase inhibitor imatinib mesylate (Gleevec) (162, 165). Germline truncations of *p19^ARF^* have also identified a specific region responsible for *Trp53* induction and suppression of *BCR/ABL1* leukemias (180).

These differences of *BCR/ABL1* driven disease in different lineages might suggest a separate cell of origin for B cell and myeloid disease, however using purified HSCs as donor cells suggests both CML and B ALL can arise from a long-term HSC cell of origin (170). In this model disease subtype is primarily influenced by the use of different isoforms of *BCR/ABL1*, with the longer p210 isoform giving rise to CML whereas a shorter isoform p185 causes B ALL. Despite this common cell of origin, the cancer-propagating “cancer stem cell” populations responsible for maintaining these diseases appear to be distinct. The CML disease was maintained by a long-term HSC population whereas B ALL was maintained by a differentiated pro B population.

The interactions of *BCR/ABL1* with other donor cell lesions has also identified additional essential mediators of disease. *Stat5a* and *Stat5b* are necessary for establishment and maintenance of malignancies derived from *BCR/ABL1* infected bone marrow (163, 167) and N-terminal truncation of *Stat5a* and *Stat5b* skews *BCR/ABL1* p210 transplants toward a B cell lineage but *BCR/ABL1* p185 transplants are unaffected (154). *BCR/ABL* p210 fails to induce disease when using *Cdk6^−/−^* bone marrow, possibly due to defects in HSC cycling in these mice (175). *BCR/ABL1* transduced bone marrow lacking three Src kinases (*Lyn^-/-^;Hck^-/-^;Fgr^-/-^*) resulted in delayed onset of B ALL compared with wild type donors however this delay was not seen for the onset of CML derived using 5-fluorouracil treated donors (158). This demonstrates a B lineage specific requirement of Src kinases for *BRC/ABL1* driven malignancies, and is consistent with synergy of pan Src kinase inhibitor CGP76030 with imatinib mesylate in treating *BCR/ABL1*-induced B ALL but not CML.

Collectively the above literature of *BCR/ABL1* malignancy models demonstrate how the genotype and developmental stage of transduced donor cells can alter disease subtype, clonogenicity and treatment response. These studies also demonstrate the value of transplantation models in defining the tumor initiating cell types of genetically similar (if not identical) but phenotypically distinct diseases.

### Modelling Disease Derived From Specific Stages of B Cell Development

One disadvantage of using HSCs or mixed populations of bone marrow as donor cells is their potential to develop a wide spectrum of malignancy lineages from undefined developmental stages. Culture systems for *in vitro* expansion of B cell progenitors (189, 194, 195) have been further refined to use B220+Kit+ fetal liver cells cultured with IL-7 on ST-2 stromal cells (168) and more recently stroma free pro B cell culture conditions have also been developed using IL-7, SCF and FLT3 ligand (196). These systems allow expansion and transduction of progenitor/precursor B cells for use in transplantation studies. In one study, pro B cells were transduced with combinations of *Bcl2*, *Myc* and *Ccnd1*, and transplanted into severe combined immunodeficient (SCID) mice. The recipients develop an immature B cell lymphoma/leukemia which infiltrated the lymph nodes, spleen, thymus and bone marrow. *Ex vivo* cultured pro B cells transduced with *Bcl2* and *Myc* were also transduced with a cDNA library and selected for continued growth in the absence of IL-7 and ST-2 stromal cells. *CCND3* and *NRAS* both rendered these cultures independent of IL-7 and ST-2 cells and accelerated *Myc/Bcl2* driven disease when transplanted into SCID mice.

Long term proliferative pre B cell lines derived from fetal liver have also been used to identify and characterize genes that cooperate with *Myc* in an *in vivo* setting (195). Tetracycline inducible vectors expressing both *Myc* and *Pim1* have demonstrated an interdependent role of these genes in allowing *in vivo* expansion of pre B cell lines, and this expansion is dependent upon continued expression of both genes (169). In a similar study, doxycycline inducible *Myc* and *Bcl2l1* (*Bcl-xL*) pre BI cells were differentiated to immature B1 cells *in vitro*, and timed CpG stimulation generated either pre BII-like or mature B1-cell lines and IgM-secreting B1 cells. Introduction of these pre BI cells into *Rag1* ^-/-^ mice gives rise to plasmablast and plasma cell hyperplasia that was reversible when doxycycline was removed (179).

This model was also used to screen a library of cDNAs in pre B cell lines to identify genes that cooperate with *Myc* (178). Cells were selected *in vitro* in the absence of stromal cells and IL7 and *in vivo* by transplantation into *Rag1* ^-/-^ immunocompromised recipients. These screens identified multiple *Myc* cooperating genes including *Exosc1*, *Rpl18a*, *Rpl35a Ndufs7*, *Cacybp*, and *Ptprcap*. Overexpression of both a full length and truncated form of *Exosc1* (a component of the RNA exosome) were validated as cooperating with *Myc* and the authors proposed the mechanism is a function of global inhibition of mRNA degradation.

Later stages of B cell development can also be reproduced *in vitro*. Propagating naïve splenic B cells on a layer of 3T3 fibroblasts expressing both *CD40L* and *BAFF* causes them to proliferate into germinal center B cell like cells called *in-vitro*-induced germinal center B (iGB) cells (190). When iGB cells were cultured with IL-4 they differentiated toward memory B cell precursors and IL-21 steered differentiation to a long-lived plasma cell phenotype. Retroviral transduction of these iGB cells with *Myc* and *Bcl2* and transplantation into sublethally irradiated syngeneic recipients led to development of aggressive DLBCL like disease (172).

It is worth noting that *in vivo* library based screens of B cell biology need not be strictly limited to readouts of malignancy. Various models of B cell development and positive/negative selection of B cells are also relevant to malignancy. The IgMb-macroself mouse strain ubiquitously expresses a superantigen that causes deletion of immature B cells through reaction of surface IgM with a “self” superantigen. Transduction of bone marrow HSPCs with retroviruses encoding a miRNA expression library, and reconstitution of macroself recipients led to selection of cells expressing *miR-148a*, which was then verified to prevent deletion of self-reactive B cells (197).

The heavy chain of the B1-8^hi^ transgenic strain has an increased affinity to the 4-Hydroxy-3-nitrophenylacetyl (NP) hapten after immunization and this model has been used to screen for modifiers of germinal center formation. Splenic B cells from B1-8^hi^ transgenic mice were stimulated with anti-CD180 antibody, transduced with a library of shRNAs and transplanted into wild-type C57BL/6 mice (198). Recipients were then immunized with NP-chicken gamma globulin and alum to stimulate germinal center formation by the transplanted cells. Comparing the ratio of shRNA transduced B cells from germinal centers to non-germinal center cells revealed selection against *Zdhhc2* shRNA, thereby demonstrating a role for this gene in productive germinal center formation. *Zdhhc2* shRNA also inhibited development of iGB cells *ex vivo*. Although these two studies do not model tumor development, they demonstrate how specific stages of B cell development can be probed using adoptive transfer of primary B cells to perform reverse genetic screens.

### CRISPR-Cas Gene Editing in B Cell Malignancy Models

Various site-specific nucleases such as zinc finger nucleases, transcription activator-like effector nucleases (TALENs) and Cas endonucleases have been adapted to targeted editing of mammalian genomes (199). Since the proof of concept of CRISPR-Cas editing in mammalian cells (200, 201) this system has gained popularity primarily because the target site specificity is determined by a short guide RNA (sgRNA) sequence that includes a ~20 nucleotide segment that is complementary to the target site sequence. This property makes CRISPR-Cas targeting constructs easier to use than zinc finger nuclease and TALEN constructs which express large open reading frames and require longer sequences of protein encoded DNA binding motifs to determine target site specificity. There can also be gene/locus specific differences between the technologies, with TALENs demonstrating greater activity in heterochromatin and Cas9 greater activity in euchromatin (202).

The short targeting sequence of CRISPR-Cas endonucleases facilitates functional genomics experiments with a similar throughput to shRNA vectors, with the additional benefit of editing target alleles permanently rather than changing gene expression by lowering mRNA levels. Like shRNAs, sgRNAs can vary in their efficiency and off target effects. Double stranded DNA breaks created by SpCas9 are primarily repaired by nonhomologous end joining (NHEJ) which is an error prone process. Consequently, some earlier versions of CRISPR-Cas editing have been shown to create imprecise lesions. Depending on the delivery system used, a subset of target sites may generate deletions of thousands of bases, complex rearrangements and crossover events (203–207). Nonetheless, technical innovations including novel Cas variants and fusions, improved sgRNA design, optimized delivery methods and allele replacement by homologous recombination have reduced off target effects, increased efficiency, improved specificity and even permitted mutation of single bases (208–225).

CRISPR-Cas genome editing has been adapted to modelling a variety of tumor types either through loss of function mutations, chromosomal engineering or gain of expression [reviewed in (226, 227)]. CRISPR-Cas models of B cell malignancy are rare although there is now an extensive literature on conditions that increase the efficiency of editing primary B cells or HSCs. Some researchers use cells from mice expressing Cas9 and transduce these with viruses expressing sgRNAs (228–233). In one of these studies, activated B cells from mice expressing a Cre inducible Cas9 IRES GFP from the *Rosa26* locus were transduced with sgRNA viruses, achieving up to 80% knockout efficiency in cultured primary B cells (231). Another study transduced lipopolysaccharide (LPS) stimulated B cells expressing Cas9 from the *Rosa26* locus with virus expressing sgRNA and a puromycin resistance marker, followed by selection with puromycin to enrich for edited cells (230). Others have used Cas9 expressing Lineage- Sca-1+ Kit+ (LSK) HSCs from bone marrow, transduced these with lentivirus sgRNA constructs, yielding edited cells of multiple hematopoietic lineages in reconstituted recipients including B cells (232). B cell specificity has also been achieved *via* the transduction of LSK HSCs bearing a *Cre* switchable Cas9 under control of a CD19-*Cre* transgene (233). The knockout efficiency of target genes in CD34+ HSPCs from human cord blood can also be improved by changing the order of delivery and the sgRNA structure, and these cells remain transplantable into immunocompromised mice after transduction (234).

Editing can also be made more efficient by delivering Cas9 as a protein, as an mRNA or as a ribonuclear protein of Cas9 precomplexed with sgRNA [reviewed in (217)]. Activating culture conditions also play a role in efficient transduction of B cells. Human peripheral blood B cells can be expanded by IL4 and CD40L cross linking and then electroporated. Electroporation with *CD19* targeting sgRNAs in combination with either Cas9 RNA or Cas9 protein, can achieve knockout of *CD19* in up to 70% of cells (235). Mouse splenic B cells activated using LPS (as a TLR4/CD180 agonist), or human peripheral blood B cells activated by anti-CD180 antibody can be edited by electroporated Cas9/sgRNA ribonuclear protein combined with adeno associated virus templates (236) or transfection of Cas9/sgRNA ribonuclear protein with ssDNA template (237). Human peripheral B cells cultured in IL-2, IL-10, IL-15, multimerized CD40 ligand and CpG oligodeoxynucleotide are efficiently edited by electroporation with CRISPR/Cas9 ribonuclear protein and adeno associated virus template constructs (238).

Although CRISPR-Cas has not been used to study lymphoma and leukemia as often as shRNAs, the technology has proven utility in validation of tumor suppressor candidates. One of the earliest proof of concept experiments used a vector expressing both Cas9 and an sgRNA against *Trp53*. When established Eμ-*Myc p19^ARF-/-^* lymphomas were transduced and transplanted into recipients, lymphoma growth *in vivo* was accelerated (138). Another model used a vector constitutively expressing Cas9 with a doxycycline inducible vector expressing *Trp53* sgRNA in Eμ-*Myc* HSCs. This yielded rapid 100% penetrant disease where both *Trp53* alleles could be found modified by a range of insertions, indels, deletions and large deletions (173). A similar model was subsequently developed using a doxycycline inducible Cas9 transgene in donor cells (183).

Three of the forward genetic studies mentioned previously in this review used similar Eμ-*Myc* fetal liver HSC transplant models to verify the tumor suppressors *Bcor* (using a vector expressing both Cas9 and sgRNA) (54, 108) and *Rfx7* and *Phip* (combining a sgRNA vector with a Cas9 transgene) (48). Eμ-*Myc* HSCs have also been used to perform CRISPR-Cas9 functional genomic screens. In one study a library of 75 sgRNAs was designed against rarely mutated genes in Burkitt’s lymphoma and cloned into a Cas9 expressing vector. Pools of up to 5 sgRNAs were then transduced into Eμ-*Myc* fetal liver cells. Transduced cells were transplanted into irradiated recipients and when lymphomas developed, sgRNAs were quantitated by PCR and sequencing; genomic editing was then measured by T7 endonuclease I mismatch assays. Of the candidate sgRNAs identified by the screen *Phip* and *Sp3* were functionally validated as tumor suppressors using the same assay (177).

Cas/sgRNA targeting has also been expanded to the regulation of transcription by fusing nuclease deficient SpCas9 to transcriptional domains that either activate transcription (referred to as CRISPRa) or inhibit transcription (referred to as CRISPRi) (239). CRISPRi constructs have modified *in vivo* growth of primary Eμ-*Myc*;*p19^ARF-/-^* lymphoma and have been used to conduct an *in vivo* screen in *Bcr-Abl* driven B ALL (134). From a 25 gene library of sgRNAs, *Chk2* was identified as a modifier of temozolomide resistance.

CRISPR-Cas has also been used to engineer translocation events in somatic cells for mouse models of cancer. One of the earliest examples was induction of the *EML4/ALK* translocations in a lung cancer model (240) and since then CRISPR-Cas induced translocations have also been adapted to hematological malignancies, with a handful of models generating a subset of B lineage malignancies alongside other subtypes. One small study using mouse donor cells introduced SpCas9 and guide RNAs that translocate *Npm1* to *Alk* in mouse fetal liver HSCs. Transplanting these into recipients lead to two cases of T lineage ALK+ anaplastic large-cell lymphoma and one case of anaplastic ALK+ large B-cell lymphoma (186). Two studies discussed in the next section have also used CRISPR-Cas editing to cause malignancies by introducing these same translocations in human HSCs (182, 185).

### Xenotransplantation Models of Human Leukemia

Human cord-blood derived HSCs have been used in a manner similar to mouse derived HSCs to generate adoptive transfer models of various hematologic malignancies. The t(11;19)(q23;p13.3) translocation produces the *MLL/ENL* (*KMT2A/MLLT1*) fusion and is found in acute leukemias of B, T and myeloid lineages. The t(9;11)(p21;q23) translocation produces the *MLL/AF9* (*KMT2A/MLLT3*) fusion and is primarily observed in childhood AML but also in a subset of ALL in children below 1 year of age. Lineage-depleted human umbilical cord blood infected with *MLL/ENL* virus gave rise to B precursor ALL when injected into sublethally irradiated NOD/LtSz-scid/scid mice (164). An *MLL/AF9* virus in the same model gives rise to a phenotypically similar B ALL and less frequently an AML like disease.

The lineage restriction of tumor initiating and tumor propagating cells was also investigated. The use of myeloid promoting suspension culture protocols skewed both transgenes toward a myeloid leukemia when injected. Transplantation of limiting cell numbers of primary tumors into secondary recipients indicated self-renewing leukemia cells were rare. Southern blots of retroviral vector integration and immunoglobulin heavy chain rearrangement suggested that leukemia initiating stem cells with germline immunoglobulin can differentiate into tumor propagating B lineage cell type having undergone immunoglobulin rearrangement. Lineage switching between B and myeloid lineages was also observed in culture.

More recently both translocations have been recreated in human HSCs using CRISPR-Cas. One model used CRISPR-Cas to induce the *MLL/AF9* (*KMT2A/MLLT3*) translocation in cultured human cord blood HSCs. Transplanting these cells into sublethally irradiated NSG mice gave rise to B ALL, acute myeloid leukemia or mixed phenotype acute leukemia, as well as mice with a mixture of AML/ALL (185). Another study induced CRISPR-Cas mediated translocations between *MLL*(*KMT2A*) and *ENL*(*MLLT1*) in human HSCs. When these cells were introduced to NSG mice, myeloid disease was most commonly observed however secondary outgrowths of B ALL were also identified (182).

Other B cell malignancies have also been modelled. Transducing human CD133+ HSCs with *MYC* ^T58A^ mutant and *BCL2* from a lentiviral vector gives rise to a “double hit” lymphoma like disease when transplanted into NOD.*Cg-Prkdc^scid^IL2rg^tm1Wjl^/*SzJ (NSG) mice (171). NSG mice with a humanized lymphoid compartment can be generated by injecting human fetal liver CD34+ hematopoietic progenitor cells into 1 Gy irradiated NSG recipients and these mice can serve as a model for virus induced primary effusion lymphoma caused by coinfection with Kaposi sarcoma-associated herpesvirus and Epstein-Barr virus (181).

### Rapid Generation of Germline Mutations

Historically, developing models using new germline alleles is time consuming. Traditional transgenesis by pronuclear injection of fertilized zygotes leads to unpredictable integration sites of unkown copy number. Gene targetting by homologous recombination in ES cells requires constructs with long homology arms and extensive screening and characterization of clones prior to blastocyst injection. Founder animals must then be crossed to verify germline transmission and to combine multiple alleles in sufficient animals to form experimental cohorts.

Higher throughput strategies to generating these models and cohorts have now been developed using various combinations of CRISPR-Cas, recombinase mediated cassette exchange and routine derivation of new ES cell lines from mice bearing multiple germline alleles [reviewed in (241)]. Superficially, generating null alleles by zygote injection of CRISPR-Cas components is less laborious and time consuming than generating constructs for allele replacement, targeting of ES cells and blastocyst injection. In practice however, the methods used to generate mouse strains have diversified and now vary considerably in the time taken to generate targeting constructs and/or the reliability and efficiency of allele generation.

One of the main advantages of CRISPR-Cas is that the targeting of multiple alleles simultaneously can disrupt both copies of a gene and/or disrupt multiple genes a single founder animal. Simultaneous CRISPR-Cas mutation of both germline alleles of B lymphoma tumor suppressors *Tet1* and *Tet2* have been achieved as inactivating mutations (242) and even conditional loss of function alleles (243), potentially saving generations of breeding time in order to establish mutant cohorts. The efficiency of generating floxed alleles by injection of zygotes has been improved by the use of Cas9-avidin fusion with biotin tagged replacement template DNA (223). As mentioned previously some versions of CRISPR-Cas editing can generate imprecise lesions and off target effects (203–207). As such the speed gained from generating multiple CRISPR-Cas edited alleles simultaneously may be somewhat offset by the need for backcrossing and the need to repeat experiments using mice derived from independent founders. Where greater precision of allele generation is required (e.g. null alleles are lethal) the use of newly rederived ES cell lines from mouse strains carrying four or more alleles of interest offers an alternative for rapid generation of multiallelic models (244, 245).

CRISPR-Cas also potentially allows novel approaches to increase the throughput of gene editing of many alleles simultaneously (246). Multi sgRNA alleles can be generated by placing a lox71 site downstream of a U6 promoter and then downstream of this a concatemers of alternating sgRNA sequences with loxKR3 lox sites. LoxKR3 sites cannot combine with each other but can recombine with lox71. As such expression of Cre recombinase will lead to a single recombination event where only one of the sgRNA sequences chosen at random will be expressed in each cell. This allows Cre inducible sgRNA mosaicism that can be employed to study a range of genes in a single animal, or using germline switching the generation of many sgRNA strains from a single progenitor allele.

### Temporal Control of Germline Alleles and Retroviral Constructs

Studies cited in previous sections drive specific disease subtypes through combinations of germline alleles, viral vectors and the cell types being cultured and transplanted. Additional control can also be achieved though vectors or alleles that are regulated by Cre recombinase or ligands such as doxycycline (for tetracycline regulatable promoters) and 4-hydroxytamoxifen (to regulate nuclear localization of estrogen receptor fusions) (247). Early regulatable models of lymphoma include mouse strains expressing human *MYC* from tetracycline/doxycycline regulatable promoters (248, 249) which develop T, B and myeloid lineage malignancies that regress upon doxycycline induced repression of *MYC* expression. A similar doxycycline repressible *BCL2* transgene when combined with the Eμ-*Myc* transgene gave rise to B lymphoblastic leukemia. These leukemias undergo remission upon repression of *BCL2* expression (250). Studies such as these demonstrate the continued requirement of cancer initiating oncogenes in tumor maintenance and thereby the potential of these proteins as therapeutic targets.

Similar regulation of shRNAs has also been achieved using tetracycline/doxycycline repressible germline alleles. Constitutive knockdown of the *Pax5* tumor suppressor by shRNA in combination with constitutively activated *STAT5b* causes a B ALL like disease (251). Repression of *Pax5* shRNA expression by doxycycline treatment removes a block in differentiation and when these established leukemias are transplanted into *Rag1^-/-^* recipients, doxycycline treatment causes remission of disease (251). An analogous model of tumor suppressor restoration is a mouse strain that fuses the carboxy terminus of the endogenous *Trp53* open reading frame to the estrogen receptor ligand binding domain. This fusion renders mice functionally *Trp53* null until treated with 4-hydroxytamoxifen (252). Restoring *Trp53* function in established Eμ-*Myc* lymphomas demonstrates the therapeutic potential for *Trp53* restoration (253), which is analogous to the stabilization of Trp53 by treatment with Mdm2/Mdmx inhibitors.

Constructs that express open reading frames or shRNAs from tetracycline/doxycycline regulatable promoters have been transduced into B cell lines or hematopoietic stem cells used for transplant studies (128, 254–258). Placing the expressed sequences of a retrovirus between tandem loxP sequences allows controlled expression of an invertible open reading frame cassette that is limited by lineage specific Cre expression (259). Viral vectors expressing doxycycline regulatable Cas9 (260) or sgRNAs (173) have also been developed. Aside from the previously mentioned strains expressing Cas9 under control of Cre recombinase (228, 233), several mouse strains have also been generated that place Cas9 under the control of tetracycline regulatable promoters (183, 261, 262). In one of these studies, the *Col1a1* locus was targetted in ES cells with a construct expressing multiple sgRNAs constitutively and a tetracycline/doxycyline regulatable Cas9 expression cassette. The resulting mice were crossed to mice with a reverse tetracycline transactivator allowing doxycycline controlled biallelic mutation of multiple genes in somatic cells using only two alleles (262).

CRISPRi gene regulation has similarly been placed under doxycycline control; Sp-dCas9 (nuclease deficient Cas9) is fused with the VP64 transactivation domain (i.e. four copies of the herpesvirus VP16 domain), the RELA p65 transactivation domain and the reverse tetracycline activation domain (263). This fusion has relatively low activity until cells are treated with doxycycline at which point the coexpression of sgRNA sequences causes activated expression of genes determined by the sgRNA target sequence.

## Conclusions and Future Directions

Human populations are highly polymorphic and this, in combination with the mutator phenotype generated by many tumours, can make it difficult to subtract the background noise of unselected passenger mutations from the evidence for driver mutations. The growing volume of human cancer genome sequencing data has created an exponentially expanding number of potential validation experiments. This in turn creates a prioritization bottleneck deciding which combinations of mutations should be tested and the developmental stages in which they should be modeled.

Forward genetics in mice can aid this prioritization by cross species comparative genomics i.e. identifying the overlap of mutation spectra identified between human and mouse cohorts. There is a tradeoff between the extent to which forward genetic screens recapitulate human disease and the throughput of mutation detection. Exome sequencing arguably better mimics human tumor exomes, however insertional mutagenesis screens better represent the effects of translocation/copy number/non-coding mutations and are relatively lower cost and higher yield in terms of the number of driver mutations identified per mouse. This cost benefit analysis becomes relevant when considering the statistical limits of proving selection of non-coding point mutations from WGS of human cohorts. Insertional mutagenesis models also have greater statistical power to identify cooperating mutations, especially when sequencing coverage is sufficient to identify subclonal mutations.

Forward genetic screens in mice also have limitations. In some cases, the expected homologs of human drivers are not identified because functionally equivalent mutations that are specific to mice are identified instead. Another limitation of both forward and reverse genetic models is that the time frame for development of malignancies in mice is far shorter than observed in human lifespans. Consequently, predisposing mutations must be introduced and this in turn increases the chance of multiple clones arising independently in a single animal. Furthermore, Cre expressing strains used in some studies (such as Cd19 and Aicda) may replace the endogenous gene thereby altering the dosage of crucial B cell development genes.

Since the emphasis of this review is experimental approaches with higher throughput to identify and validate large numbers of candidate genes, for the most part we have not covered reverse genetic models that exclusively employ cohorts generated by germline alleles, which is a vast literature in and of itself. Rapid model prototyping using transplantation of virus transduced cells reduces the commitment of resources for initial testing of candidate genes. This allows more genes to be tested before robust germline models are designed and, in a handful of instances, allows for high throughput screens of dozens or even hundreds of candidate genes within a single cohort. This approach does, however, lack the precision of inducible targeted germline alleles and the dosage of transduced constructs can be highly variable and potentially reach expression levels not observed in human cells. Many cancer genes have physiological roles that are highly sensitive to dosage, and non-physiological expression levels from vectors may block or drive differentiation, sensitize cells to apoptosis or alter proliferation in a manner not typically observed as a result of somatic mutations.

Despite the number of labs working with B cell transplantation models, very few have published functional genomic screens where selection takes place *in vivo*. *In vivo* functional genomic screens are arguably a uniquely powerful application of adoptive transfer protocols, however in writing this review we only identified a limited number of studies using libraries of ORFs, shRNAs or gRNAs in B cell malignancy models. This would suggest that there is unrealized potential for more screens and/or that there are technical barriers to the reproducible identification of constructs selected in parallel. Functional genomic screens are a tradeoff between increasing the number of constructs screened whilst controlling for the stochasticity of selection of each construct relative to others in the library. B cell survival, migration, selection, differentiation and expansion are highly stochastic processes that may obscure selection in complex libraries. One approach to controlling for this stochasticity employed by several researchers is screening smaller pools of limited numbers of constructs to make selection of individual constructs easier to observe. Another approach to increasing confidence in the set of candidates identified in a screen is performing parallel screens using both shRNAs and sgRNAs to find genes that are selected by methodologies (264).

In aggregate, the studies discussed in this review suggest several lines of research that are ripe for further innovation. The literature of transplantation-based models is dominated by the use of Eμ-*Myc* fetal liver HSCs as donor cells (often in combination with mutation of *Trp53* or the *Cdkn2a* locus). Transplantation models could easily be diversified through the use of different germline lesions or culturing and transplanting later stages of B cell development. Many of the studies we discuss have successfully cultured and transduced primary B cells with high efficiency, though currently only a handful have used transplantation of transduced B cells as the basis of lymphoma models.

Another promising direction for future development is the use of humanized models of human HSCs transplanted into immunodeficient mice. Immunodeficient host strains vary considerably in their engraftment potential, and although most are missing later stages of B cell development, they can recapitulate earlier stages of lymphoid development and have formed the basis of useful models for lymphoblastic leukemias. Immunodeficient mouse strains have various defects in the hematopoietic microenvironment and/or stromal cell proteins that are incompatible with their human targets and this can inhibit engraftment. For instance, IL2 receptor common gamma chain null mice (including NSG mice) lack lymphoid tissue inducer cells and innate lymphoid cells and do not express HLA molecules on thymic epithelia thereby preventing MHC restriction of T cells. Some models also develop graft-versus-host disease. There now is a growing literature of newer humanized models that can better recapitulate all aspects of human hematopoiesis (265–268). Newer strains compensate for these deficiencies or enhance engraftment by transgenic expression of human proteins (including SCF, GM-CSF, IL3, SIRPA, HLA, B2M, TPO and TSLP). Other deficiencies can be partly compensated by co-engraftment of human HSCs with human mesenchymal stem/stromal cells. Although this review has mostly emphasized the use of models dissecting the biology and genetics of B cell malignancies, there is likely unrealized potential for transplant models in preclinical studies of treatment efficacy and/or identification of novel therapeutic targets.

Modelling the antigenic context of B cell malignancies is also rarely addressed in the literature. The role of antigenic stimulation or tolerization by commensal flora is frequently overlooked and SPF or germ free conditions may not accurately represent the microbiome of human cancer patients, particularly where disease is influenced by a combination of normal gut flora and infectious agents, as is the case with *H.pylori* infection in MALT lymphoma (269).

Identification of human cancer drivers and the cell types that are most responsible for tumor initiation and propagation are critical foundations for the discovery of novel targeted therapies. The “post-genomic era” with seemingly limitless genomic data to interrogate has brought its own challenges of how to prioritize the use of this data. Modeling large numbers of mutations over the many stages of B cell development is a vast undertaking, particularly when differentiating between genes responsible for tumor initiation and maintenance and when testing potential targets across diverse genetic contexts of a single disease subtype. As such even the highest throughput experimental approaches discussed here can benefit from the parallel use of complementary methods.

## Author Contributions

All authors listed have made substantial, direct, and intellectual contribution to the work and approved it for publication.

## Funding

JD and AU were supported by MRC programme grant MC_A652_5PZ20.

## Conflict of Interest

The authors declare that the research was conducted in the absence of any commercial or financial relationships that could be construed as a potential conflict of interest.

## Publisher’s Note

All claims expressed in this article are solely those of the authors and do not necessarily represent those of their affiliated organizations, or those of the publisher, the editors and the reviewers. Any product that may be evaluated in this article, or claim that may be made by its manufacturer, is not guaranteed or endorsed by the publisher.
